# Immunology reshapes neuroscience, and neuroscience reshapes immunology

**DOI:** 10.1016/j.fmre.2024.01.003

**Published:** 2024-01-18

**Authors:** Bo Peng, Yanxia Rao, Yun Wang, Shumin Duan, Hai Qi, Jing Yang, Hongliang Zhang

**Affiliations:** a Department of Neurosurgery, Huashan Hospital, Institute for Translational Brain Research, State Key Laboratory of Medical Neurobiology, MOE Frontiers Center for Brain Science, MOE Innovative Center for New Drug Development of Immune Inflammatory Diseases, Fudan University, Shanghai 200040, China; b Department of Laboratory Animal Science, MOE Frontiers Center for Brain Science, Fudan University, Shanghai 200032, China; c Neuroscience Research Institute and Department of Neurobiology, School of Basic Medical Sciences, Key Laboratory for Neuroscience, Ministry of Education/National Health Commission and State Key Laboratory of Natural and Biomimetic Drugs, Peking University, Beijing 100083, China; d PKU-IDG/McGovern Institute for Brain Research, Peking University, Beijing 100871, China; e Faculty of Medicine and Pharmaceutical Sciences, Zhejiang University, Hangzhou 310014, China; f School of Medicine, Tsinghua University, Beijing 100084, China; g IDG/McGovern Institute for Brain Research, Center for Life Sciences, School of Life Sciences, Peking University, Beijing 100871, China; h Chinese Institute for Brain Research, Beijing 102206, China; i Department of Life Sciences, National Natural Science Foundation of China, Beijing 100085, China

As a result of evolution, our body has evolved into a sophisticated system in which various tissues and organs cooperate in a highly orchestrated manner. Few tissues or organs operate in isolation. The brain serves as the command center of our body and is relatively isolated from the peripheral system due to the presence of the blood-brain barrier (BBB). This barrier prevents direct and extensive interaction between blood cells, plasma molecules, and brain cells, contributing to the long-standing perception of the brain as an “immune-privileged” area. Consequently, neuroscientists and immunologists historically conducted separate research with minimal overlap between these two disciplines. However, this perspective has undergone reconsideration over the past few decades.

The brain *per se* is not entirely “immune-privileged”, as not all brain regions are shielded by the BBB. Several BBB-devoid brain regions lack pericytes and astrocyte endfeet-wrapped vasculature, including the pineal gland, median eminence, subcommisural organ, neurohypophysis, subfornical organ, area postrema, and organum vasculosum of the lamina terminalis [1]. This unique feature allows blood cells and plasma molecules to directly interact with these brain regions, enabling them to sense and modulate the body's homeostasis directly. Even in BBB-containing brain regions, circulating cells can infiltrate and influence brain function under physiological conditions [2]. Furthermore, the brain harbors resident immune cells, such as microglia and border-associated macrophages (BAMs), both of which are yolk sac-derived myeloid cells. Microglia, characterized by IBA1^+^ P2Y12^+^ TMEM119^+^MRC1^−^ and MS4A7^−^ are the predominant myeloid cells in the brain parenchyma. BAMs, identified by IBA1^+^ P2Y12^−^ TMEM119^−^ MRC1^+^ and MS4A7^+^, are located in the meninges, choroid plexus, and perivascular space (also known as Virchow-Robin space). As the majority of resident myeloid cells in the brain, microglia play vital roles under both physiological and pathological conditions. Recent studies have also revealed mutual regulation between microglial activity and neuronal activity [3], [4], [5], [6], [7], [8]. Additionally, nearly all neurological diseases are linked to immune responses, involving either local microglia or peripheral circulating cells [9], [10], [11], [12].

This special issue of *Fundamental Research* features the frontiers of neuroimmune interplay and regulation, comprising a total of nine review or perspective articles. The topics covered in this special issue encompass a wide range of interests related to the physiological and pathological functions of neuroimmune regulation. From the perspective of the nervous system, the issue includes discussions on the interaction between the nervous system and immune system [13], the emerging strategy for treating neurological disorders through microglia replacement [14], the regulatory roles of meningeal lymphatic vasculature [15], and the relatively overlooked brain cell type, fibroblast [16]. From the viewpoint of the peripheral system, this special issue also delves into the contribution of the peripheral autoimmune system [17] and bone marrow cells [18] to the brain, as well as the regulatory roles in the pancreas [19] and skin [20]. Additionally, a comprehensive review summarizes the current state and future prospects of neuroimmunology [21]. Together, these papers provide a comprehensive overview of the intricate and highly orchestrated neuroimmune system.

## Declaration of competing interest

The authors declare that they have no conflicts of interest in this work.
